# Complex Coacervate Materials as Artificial Cells

**DOI:** 10.1021/accountsmr.2c00239

**Published:** 2023-02-13

**Authors:** Alexander B. Cook, Sebastian Novosedlik, Jan C. M. van Hest

**Affiliations:** † Bio-Organic Chemistry, Institute for Complex Molecular Systems, Eindhoven University of Technology, Helix, P.O. Box 513, 5600 MB Eindhoven, The Netherlands

## Abstract

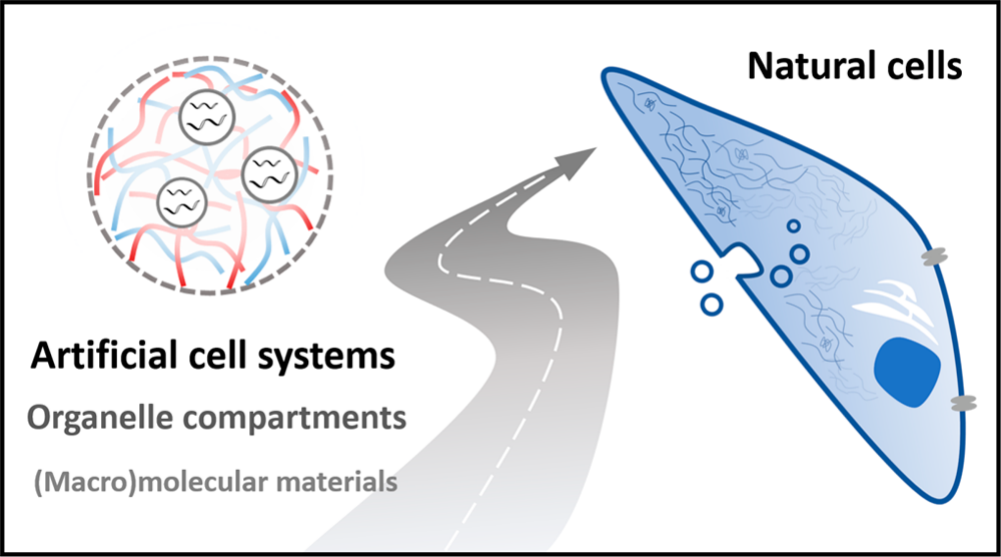

Cells have evolved to be self-sustaining compartmentalized systems that consist of many thousands of biomolecules and metabolites interacting in complex cycles and reaction networks. Numerous subtle intricacies of these self-assembled structures are still largely unknown. The importance of liquid–liquid phase separation (both membraneless and membrane bound) is, however, recognized as playing an important role in achieving biological function that is controlled in time and space. Reconstituting biochemical reactions *in vitro* has been a success of the last decades, for example, establishment of the minimal set of enzymes and nutrients able to replicate cellular activities like the *in vitro* transcription translation of genes to proteins. Further than this though, artificial cell research has the aim of combining synthetic materials and nonliving macromolecules into ordered assemblies with the ability to carry out more complex and ambitious cell-like functions. These activities can provide insights into fundamental cell processes in simplified and idealized systems but could also have an applied impact in synthetic biology and biotechnology in the future. To date, strategies for the bottom-up fabrication of micrometer scale life-like artificial cells have included stabilized water-in-oil droplets, giant unilamellar vesicles (GUV’s), hydrogels, and complex coacervates. Water-in-oil droplets are a valuable and easy to produce model system for studying cell-like processes; however, the lack of a crowded interior can limit these artificial cells in mimicking life more closely. Similarly membrane stabilized vesicles, such as GUV’s, have the additional membrane feature of cells but still lack a macromolecularly crowded cytoplasm. Hydrogel-based artificial cells have a macromolecularly dense interior (although cross-linked) that better mimics cells, in addition to mechanical properties more similar to the viscoelasticity seen in cells but could be seen as being not dynamic in nature and limiting to the diffusion of biomolecules. On the other hand, liquid–liquid phase separated complex coacervates are an ideal platform for artificial cells as they can most accurately mimic the crowded, viscous, highly charged nature of the eukaryotic cytoplasm. Other important key features that researchers in the field target include stabilizing semipermeable membranes, compartmentalization, information transfer/communication, motility, and metabolism/growth. In this Account, we will briefly cover aspects of coacervation theory and then outline key cases of synthetic coacervate materials used as artificial cells (ranging from polypeptides, modified polysaccharides, polyacrylates, and polymethacrylates, and allyl polymers), finishing with envisioned opportunities and potential applications for coacervate artificial cells moving forward.

## 1 Introduction

The development of artificial cells as interactive systems that mimic features and functions of life is a significant research challenge with the potential for far reaching impact.^1,2^ Through the collaboration of researchers from multiple scientific disciplines including organic chemistry, synthetic biology, and bioengineering, progress has recently been made toward this challenging goal. Artificial cell research will improve our fundamental understanding of biological systems, offer insights into how life could have evolved on early earth, and open the door to a wide range of possible health technologies from therapeutics to tissue engineering and organoid development.^3,4^

Bottom up assembly of synthetic macromolecules, biological components such as nucleic acids and proteins, and other small molecules can allow for the production of capsules or droplets with cell-like properties.^5,6^ This molecular approach complements top-down artificial cell fabrication, which utilizes parts of deconstructed eukaryotic cells (or even whole prokaryotes) as functional building blocks, included in cell-sized synthetic scaffolds. Irrespective of the fabrication approach taken, synthetic cells aim to mimic several features of biological cells: (i) Compartmentalization has been shown to be essential both for the individual structure of the cell and for many intracellular processes which occur exclusively within specific organelles. (ii) Information processing is performed in a number of ways, from biomolecular genetic material reproduction to the application of chemical signals. (iii) Cell adaptability and motility are important traits to mimic with artificial cells. (iv) Finally, the more challenging features to mimic growth and division–which are beginning to be investigated.

**Figure 1:**
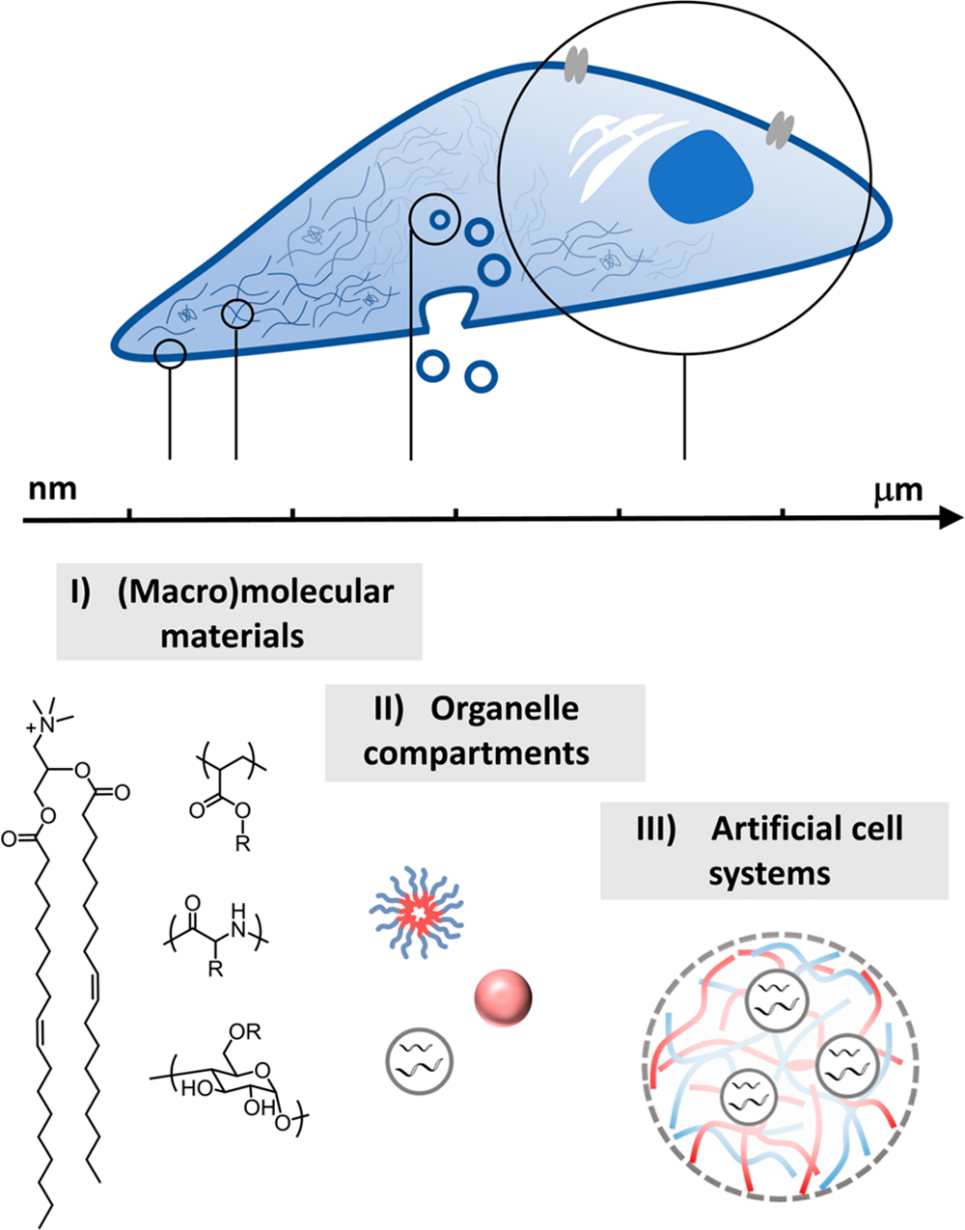
General schematic showing approaches to fabricate coacervate-based artificial cell systems, i) (macro)molecular materials used for both liquid–liquid phase separated coacervate droplets and membranes, ii) strategies to include subcellular compartments through further phase separation or inclusion of polymersomes or other nanoscale particles, and iii) assembly of all components to form micrometer-sized hierarchical coacervate-based artificial cells.

The field of artificial cell research has seen rapid growth, with research groups taking different approaches to mimicking life-like characteristics of cells with synthetic materials (overview Figure 1). Possibly the most studied systems are formed from the self-assembly of lipid giant unilamellar vesicles (GUV) which offer uniform aqueous droplets with sizes similar to those of cells surrounded by a lipid membrane.^6−8^ Advantages of these systems are ability to include protein synthesis machinery and other delicate supramolecular processes due to the idealized aqueous environment. Various groups have shown elegant examples of dynamic nonequilibrium self-assembly inside aqueous droplets as well as processing of genetic information and production of proteins.^9^ The downsides of GUVs and aqueous droplets as artificial cells are the limited resemblance of the aqueous lumen to the viscous highly crowded cytosol of natural cells. Other artificial cell systems take advantage of droplets (from both water-in-oil and water-in-water droplets through emulsion templating, and also microfluidic preparation methods) to perform compartmentalized reactions and cytoskeleton mimicking self-assembly processes.^8^ Hydrogel-based artificial cells can have physical properties more similar to those of cells than of droplets,^10^ and recent examples have shown *in vitro* translation transcription (IVTT) expression of proteins inside micrometer-sized hydrogels.^11^ The final main class of artificial cell materials are based on liquid–liquid phase separated (LLPS) droplets or complex coacervates, which mimic the viscous, dynamic, and electrolyte-rich nature of the cell interior and are in fact also naturally observed in living cells. These macromolecular condensates are important for many intracellular processes and membraneless organelles, including the formation of RNA rich phases like nuclei and stress/p granules.^12,13^ The fabrication of artificial cells from macromolecular coacervates is therefore a highly relevant method for mimicking biology. Indeed, coacervate droplets (both with and without outer membranes) have been studied by various groups with recent successes in elucidating RNA localization as well as duplex thermodynamics and mechanisms, along with incorporation of bacterial systems for dynamic/growing artificial cells.^14,15^

In this Account, we will highlight new developments of synthetic and semisynthetic macromolecular coacervate materials in mimicking cellular systems. We start with briefly discussing some fundamental aspects of coacervate formation, including an overview of the synthetic macromolecules used for coacervate artificial cells to date. We then consider the key features of natural cells and how coacervates have mimicked these, referring to key examples from literature and our group’s own research. Excellent recent reviews on hydrogels, vesicles, and aqueous droplets as artificial cells can be found, so these will not be considered here.^10,16^ Finally, we mention some areas of research where coacervate-based artificial cells can continue to push the boundaries of biomimetic systems, to achieve increasingly complex and dynamic life-like materials.

## 2 Coacervate Cell Models

**Figure 2:**
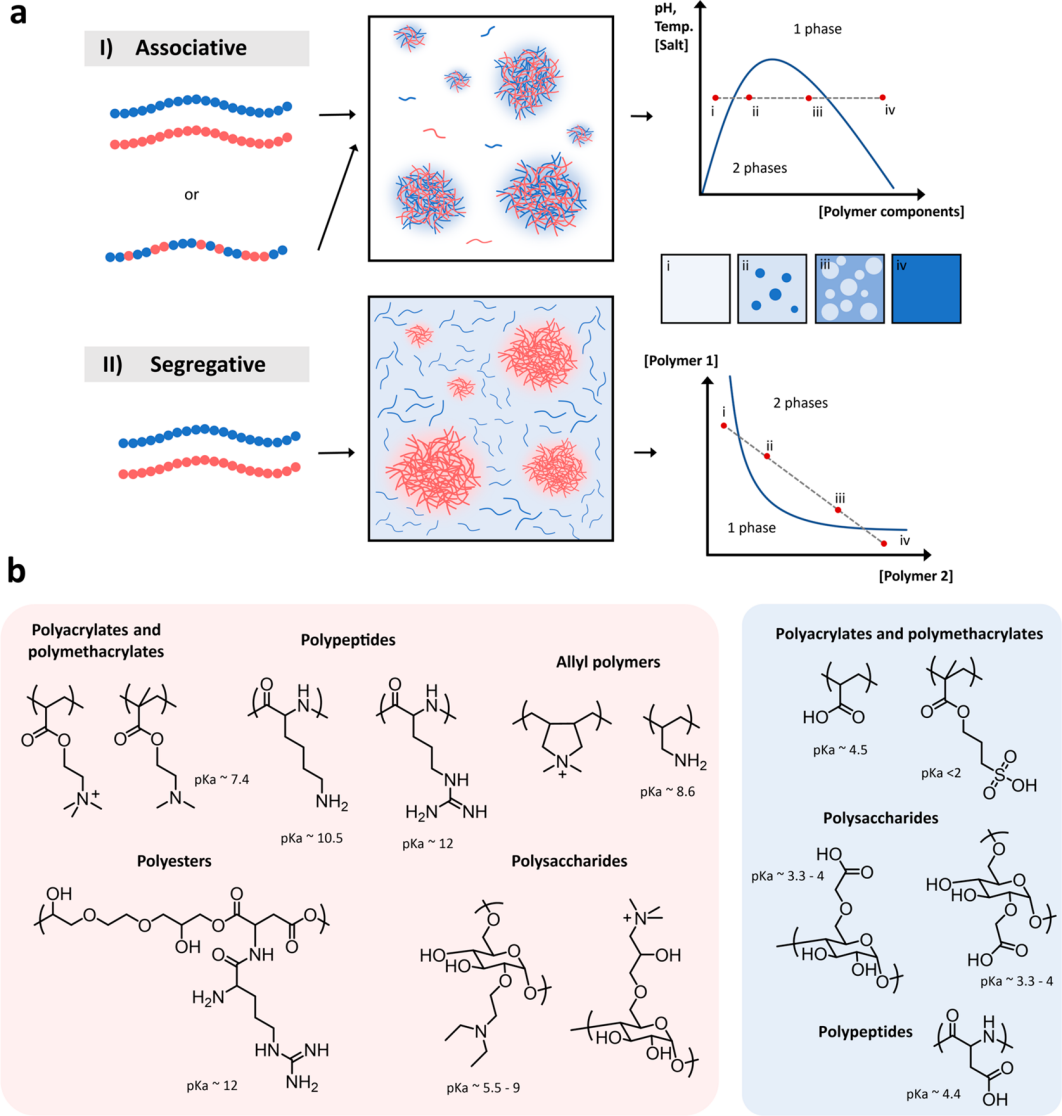
Schematic overview representing liquid–liquid phase separation of macromolecular solutions. a) Representation of associative and segregative coacervation, on the macromolecular level, with corresponding simplified phase diagrams showing one phase regions, two phase regions and tie-lines. b) Selected chemical structures of synthetic macromolecules applied as complex coacervate-based artificial cell systems.

Solutions of macromolecules in aqueous conditions can form distinct polymer dense droplets (see Figure 2).^17^ This phase separation can also be classified as aggregative or segregative depending on whether the macromolecules involved predominately exist in one phase together or separate phases and also depends heavily on conditions such as pH, salt concentration, and temperature. When these condensates are formed from two oppositely charged polymers, they are termed complex coacervates, or from one self-condensed ampholytic polymer–simple coacervates. Complex coacervates were first observed in the early 1900s, and their remarkable resemblance to the cytoplasm of cells was already mentioned. Oparin already in 1953 described coacervate droplets as an early form of a cell with potential to help explain the origin of life on earth.^18^ More recently, the use of complex coacervates to study and model artificial cells has been not only rediscovered, with particular interest in protein and RNA function in these liquid condensates, but also extended to other aspects of cellular function such as motility and signaling.

Both associative and segregative coacervation can be represented with phase diagrams, where variation of two specific conditions of the coacervate formation is combined with the observation of one/two phases on the plot (Figure 2a). The coexistence line on the plot shows the boundary between one and two phases. The diagram is a useful tool to explain, and sometimes predict, whether a system with certain polymer concentrations (or other conditions) will be phase separated or not, or the volume fractions of the two phases.

The synthetic polymer materials so far investigated as complex coacervate-forming systems include charged macromolecules in three main categories: modified polysaccharides, polypeptides, and synthetic polymers (Figure 2b). While not covered in this review, charged surfactants have also been studied in coacervation extensively. The benefits of polysaccharide systems include the high number of hydroxy functional groups able to be modified with charged moieties, natural sugar polymer backbone, typically highly water-soluble polymers even before modification with charged groups, and the possibility to synthesize libraries of charged polymers from one starting polysaccharide. Our group has used this strategy for the formation of cell-like coacervate droplets from the charged polymers carboxymethyl amylose (CM-Am) and trimethylammonium(2-hydroxypropyl)amylose (quaternized amylose, Q-Am).^19^ The group of Stephen Mann, and others, have utilized dextran polysaccharides to great effect (in particular carboxymethyl dextran, and diethylaminoethyl (DEAE) dextran).^14,20^

Polypeptides have also been extensively studied as complex coacervate-forming macromolecules.^21^ Advantages of synthetic polypeptides include the use of natural amino acids as building blocks, variability of side chain functional groups, and possibility for growing, including active droplets.^22−25^ Synthetic polymers are beginning to be used increasingly for artificial cell assembly.^26^ Their use allows for facile variation of charge density and molecular weight, access to alternative charge functional groups, architectures, and incorporation of aspects of natural systems through bioconjugation strategies.^27−31^ Charge density along the polyelectrolyte backbone has been shown to play a significant role in enzymatic processes inside coacervate droplets. Synthetic polymer chemistry and peptide chemistry allow design of libraries made of structures with varying charge densities.^32−34^ Finally, combinations of polysaccharides, polypeptides and synthetic polymers, with other natural multivalent anions such as DNA/ATP/hyaluronic acid/heparin are also being studied as coacervate artificial cell systems; however, these will be covered only briefly in this review.

## 3 Life-like System Features

In order to mimic living systems, it is useful to break the criteria down into the minimal necessary features. The following sections cover key aspects of life-like systems which can be incorporated in coacervate-based artificial cells: membranes, (hierarchical) compartmentalization, communication, motility, and metabolism and growth.

### 3.1 Membranes

Although coacervate droplets without an outer membrane are able to act as micro/macroscale condensates for dynamic processes involving facile exchange of molecules, both coalescence and nonselective uptake of molecules can sometimes hinder their use as artificial cell systems. Living cells control the exchange of nutrients and waste with the environment through a semi permeable lipid bilayer membrane.^35^ Necessary biomolecules are retained while certain small molecules exchange freely with the exterior environment, with more complex systems evolving with membrane receptors and ion channels to allow signaling and other tasks. Membranes on the coacervate droplet cell mimics can be created with a variety of molecules from lipids, synthetic block copolymers, protein conjugates, synthetic peptides, or even lysed cell membranes. In addition to amphiphile membrane formation, particle-based membranization and stabilization of cell-sized droplets is an interesting approach and could allow incorporation of different functionalities at the droplet surface.^36,37^ The coupling of membranes with coacervate systems has progressed coacervates to be more life-like by allowing semipermeable diffusion into the droplet, while also increasing stability to coalescence.

**Figure 3:**
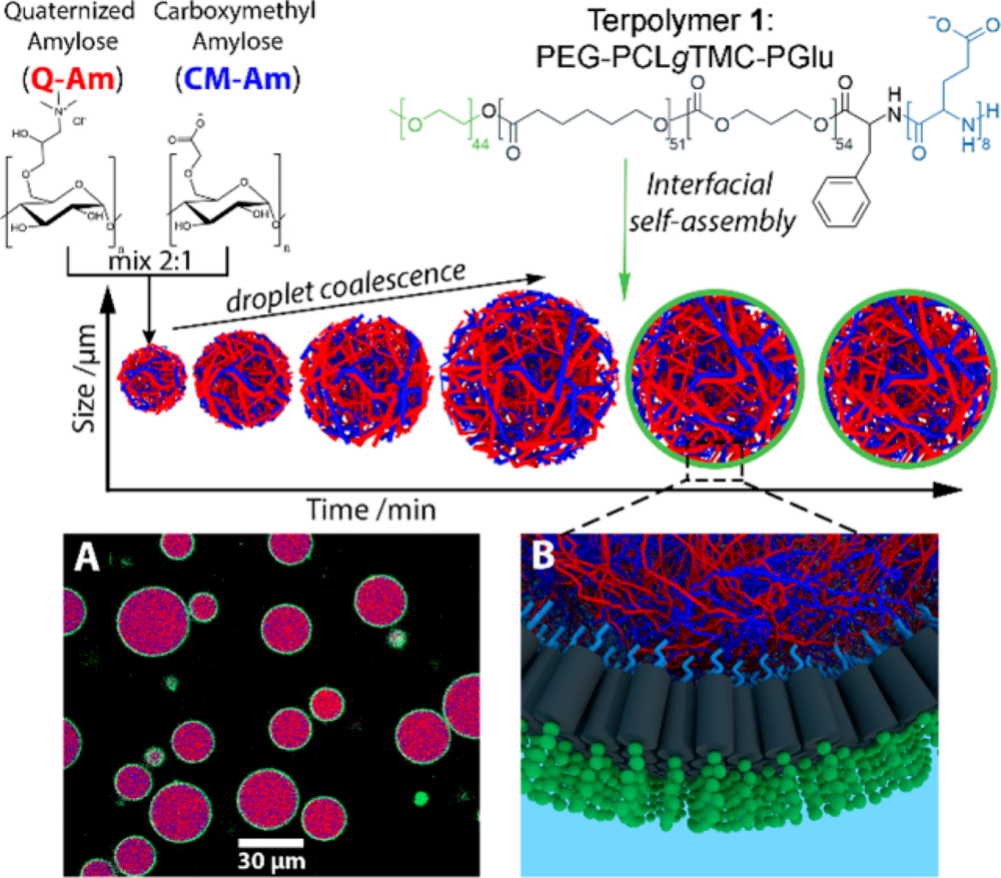
Synthetic block copolymer membrane PEG-(PCLgTMC)-PGlu and its assembly on the surface of liquid–liquid phase separated droplets, as artificial cell models. A) Confocal micrograph showing coacervate core and membrane interface. B) 3D model representation of assembled structure. Reproduced with permission from ref (19). Copyright 2017 American Chemical Society. Distributed under a Creative Commons Attribution Non-Commercial No Derivative Works 4.0 Usage Agreement.

Our group developed a triblock terpolymer for the interfacial stabilization of cell-sized coacervate microdroplets (Figure 3).^19^ The block copolymer consisted of a hydrophilic steric stabilizing poly(ethylene glycol) block, a hydrophobic poly(caprolactone-gradient-trimethylene carbonate), and an anionic poly(glutamic acid) block to facilitate anchoring to the net positively charged coacervate (PEG-(PCL*g*TMC)-PGlu). The particular design of this polymer allows a balance among electrostatically driven coacervate attraction, hydrophilic surface stabilization, and hydrophobic membrane self-association. Membranized artificial cells allow for distinct populations to be present, and therefore with the addition encapsulated enzymes, they can enable the study of complex multicomponent processes on extended time scales.

Mann and colleagues pioneered the use of coacervate cell mimic materials in 2011;^38^ in early studies the group employed fatty acids as membranes.^20^ Sodium oleate fatty acid spontaneously self-assembled at the interface of poly(diallydimethylammonium chloride) (PDDA)–adenosine triphosphate (ATP) coacervates, as well as poly(lysine)–oligonucleotide coacervates.^20^ The membranes were multilamellar and mediated selective uptake of small molecules and large (bio)molecules. The group has more recently investigated lysed cell membranes as reconstituted membranes in artificial cell systems (from both erythrocytes and bacterial cells).^14,39^ The Keating group assembled phospholipid membranes around both synthetic polymer-based and protein-based coacervate droplets through a lipid thin-film resolvation method.^40^ The membrane composition could be varied due to formulation from commercial synthetic phospholipids (1,2-dioleoyl-*sn*-glycero-3-phosphatidylcholine (DOPC), 1,2-dioleoyl-*sn*-glycero-3-phosphotidylethanolamine (DOPE), and 1,2-dioleoyl-*sn*-glycero-3-phosphotidylserine (DOPS), DOPE-PEG 2 kDa), highlighting the modular nature of their system.

### 3.2 Hierarchical Compartmentalization

Living cells rely on compartmentalization to function. For example, the genome is compartmentalized in the eukaryotic cell nuclei through the biomolecular condensate of DNA with histone proteins, and along with the nuclear envelope and nuclear matrix, maintains the integrity of genes as well as helps to regulate gene expression. This key organelle feature of cell systems is an interesting target for synthetic mimics and allows the study of complex multicomponent kinetics and cascade processes.^41−44^

**Figure 4:**
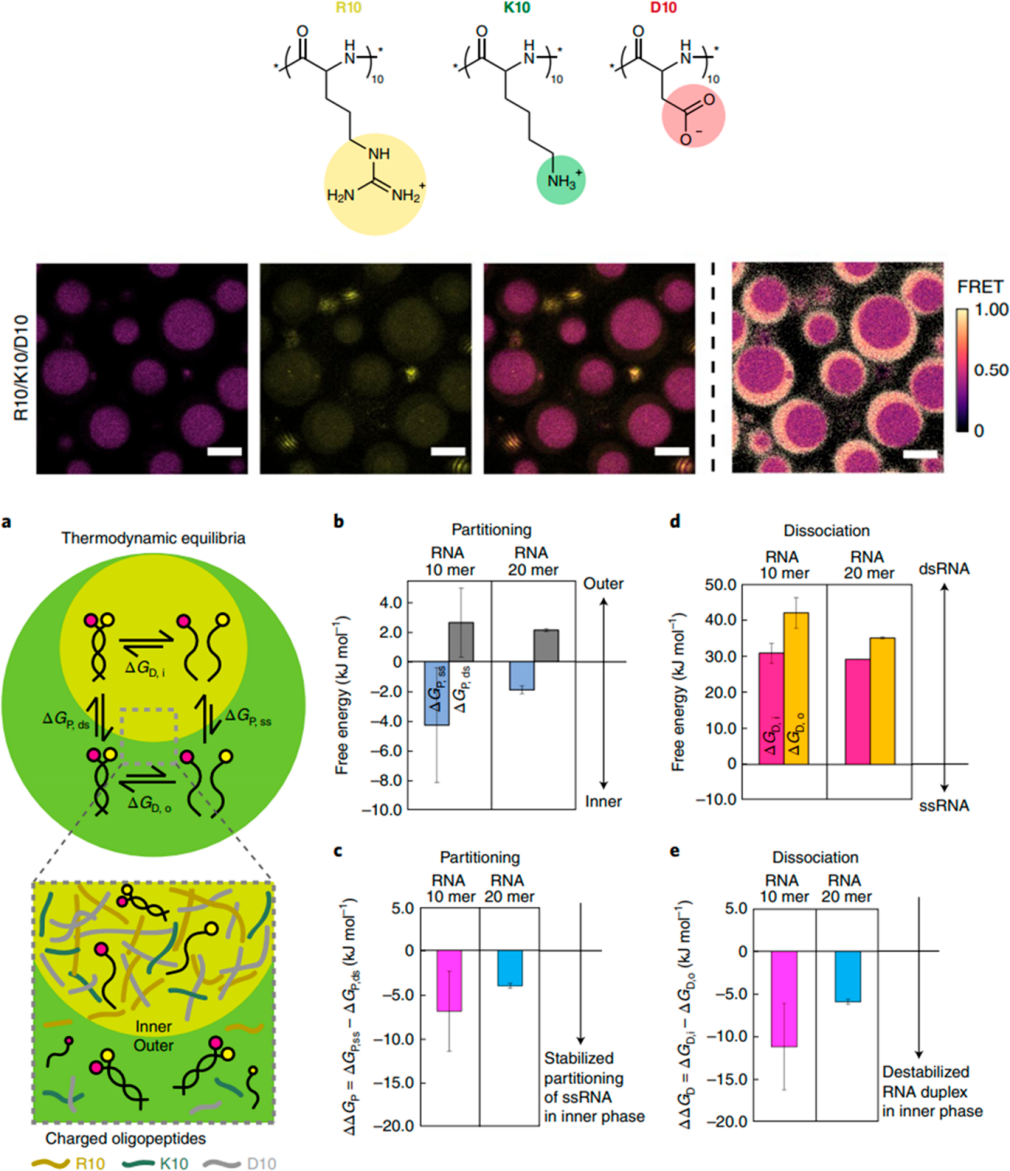
Polypeptide-based multiphase coacervates with phase specific RNA accumulation. Chemical structures of peptides studied and Förster Resonance Energy Transfer (FRET) microscopy images with RNA duplex 10mer with each strand bearing either Cy3 or Cy5 fluorophore. a) Overall thermodynamic equilibria schematic showing the proposed dynamics of the system. b), c) Quantification of partitioning thermodynamic parameters from FRET data analysis. d), e) Quantification of dissociation thermodynamic parameters from FRET data analysis. Reproduced with permission from ref (15). Copyright 2022 The Authors.

Recently, Choi et al. used short polypeptides to form multiphase coacervates and investigated the kinetics of RNA duplex formation in these different but neighboring phases (Figure 4).^15^ The peptide materials, decapeptides of arginine, lysine, and aspartic acid, were synthesized by solid phase peptide synthesis and, when combined, formed cell-sized droplets with an inner phase of higher peptide density than that of the outer phase (both phases contained all three polypeptides). Interestingly, this led to differences in RNA partitioning and RNA dissociation in the different phases, with higher amounts of duplex RNA in the outer phase, and offers insights into how membraneless organelles are able to stabilize or destabilize nucleic acids in specific compartments without the additional help of particular proteins or other binding molecules.

**Figure 5:**
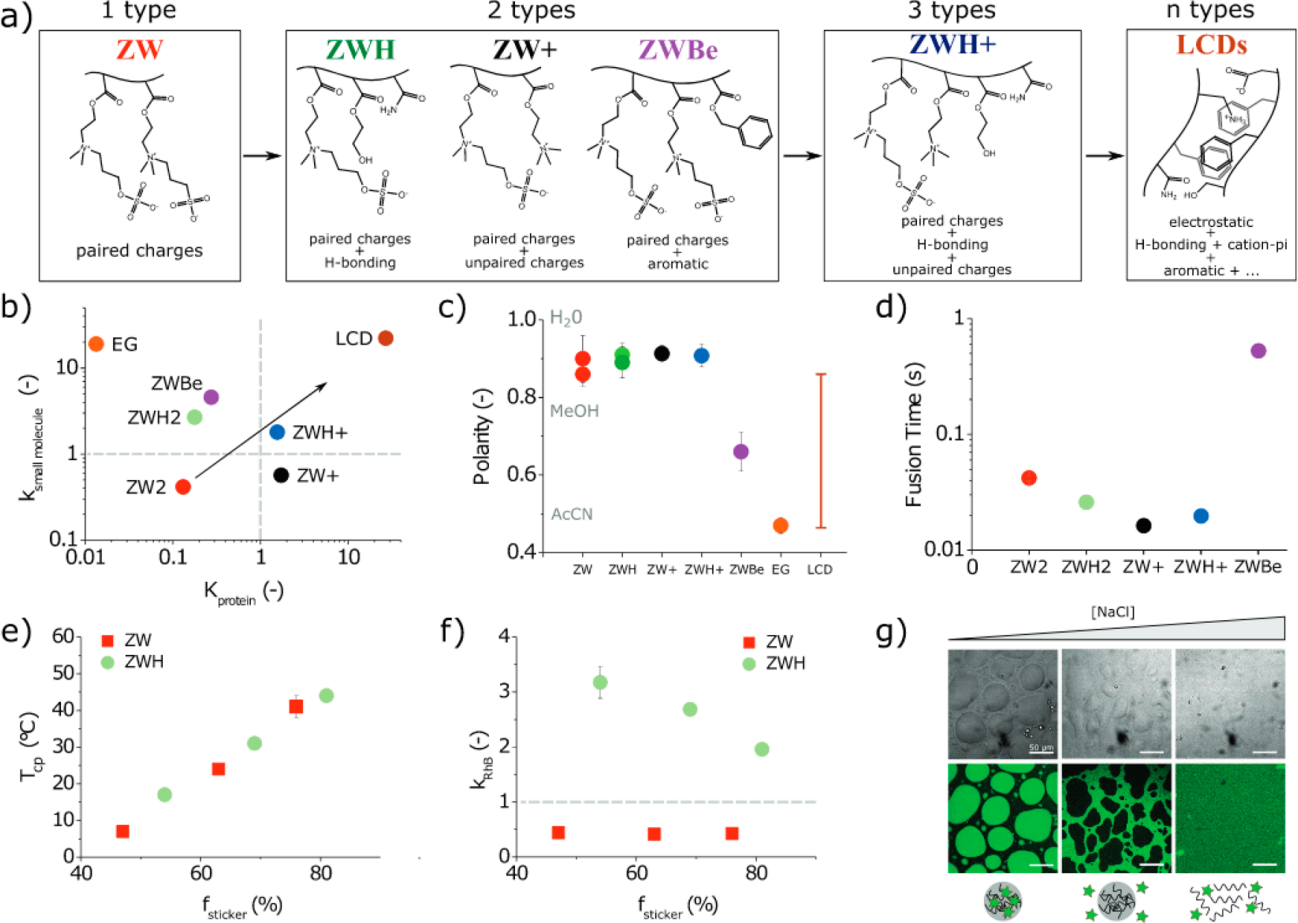
a) Synthetic polymers for the recruitment of small molecules and biomolecules. b) Partitioning coefficients of rhodamine B and BSA. c) Apparent polarity of coacervate droplets with respect to water. d) Fusion times of various polymer droplets. e), f) Cloud point of coacervate droplets varied by monomer composition. g) Salt dependence of FITC-BSA uptake and coacervate disassembly. Reproduced with permission from ref (26). Copyright 2021 The Author(s) distributed under a Creative Commons Attribution License 4.0 (CC BY-NC).

Multiphase coacervate droplets have been shown to form over a wide range of coacervate materials (both synthetic and natural, from poly(acrylic acid), ssDNA, and ATP, to poly(trimethyllysine), poly(allylamine), and DEAE-dextran) by, for example, the group of Spruijt.^45^ Coacervates formed multilayer arrangements if the coacervate to coacervate interfacial tension was lower than the interfacial tension between the surrounding media and the coacervates. The demonstration of this concept in this elegant article highlights how multiphase coacervates can be rationally designed from their monomer compositions and could be an interesting area for further research. If we consider coacervate systems formed solely from synthetic polymers, then there are fewer examples of hierarchical multiphase compartmentalization. One elegant example from Capasso Palmiero et al. shows how the modularity of synthetic polymers can be used to screen for a wide range of life-like coacervate properties (Figure 5).^26^ The authors copolymerized the zwitterionic monomers sulfabetaine methacrylate and sulfobetaine methacrylate, and the resulting polymers self-associatively phase separated into liquid droplets. Incorporation of additional monomers (hydroxyethyl methacrylate, methylacrylamide, benzyl methacrylate, and trimethylammonium ethyl methacrylate), hypothesized to mimic amino acid groups present in specific attractive interactions, e.g., hydrogen bonding, pi–pi aromatic interactions, or electrostatic charge imbalances, led to simple coacervate droplets that could be tuned to uptake certain molecules of interest. In addition, the authors combined the discovered polymers to form multiphase droplets as primitive cell mimicking materials.

Further to these excellent examples of compartmentalization through additional hierarchical phase separation, we have demonstrated that this can also be achieved by inclusion of polymersomes in coacervate artificial cell systems.^42^ The encapsulation of polymersomes or other vesicles can allow for precise separation of components, and therefore enable the study of chemical systems or enzymatic networks.

### 3.3 Communication

Individual unicellular organisms and populations of cells in tissues rely on biochemical and mechanical signaling to communicate and drive collective behavior.^46^ While artificial cells remain simplified models for intricate living cell systems, they offer an opportunity to conduct studies involving signaling and reaction networks in a biomimetic compartmentalized fashion. Researchers have made progress in modeling communication behavior in both artificial cell–artificial cell systems and artificial cell–living cell systems. The mechanisms employed for artificial cell communication are varied and can be triggered by stimuli either external or internal to the system. Small molecule signaling chemicals can diffuse into and from coacervates depending on electrostatic interactions or hydrophobicity, while larger biomolecules, such as nucleic acids or proteins, can have additional physical and/or multivalent interactions making controlled uptake and excretion from coacervates challenging. In such cases, different moieties can be used to tune noncovalent affinities, for example, nucleic acid strands, or histag NTA chemistry can be used to promote coacervate uptake. Future applications of this type of research could include use of artificial cells to regulate antimicrobial activity, inducing cell differentiation, rescuing particular cell functions, inducing cell death, and use in biohybrid synthetic tissues.^3^

**Figure 6:**
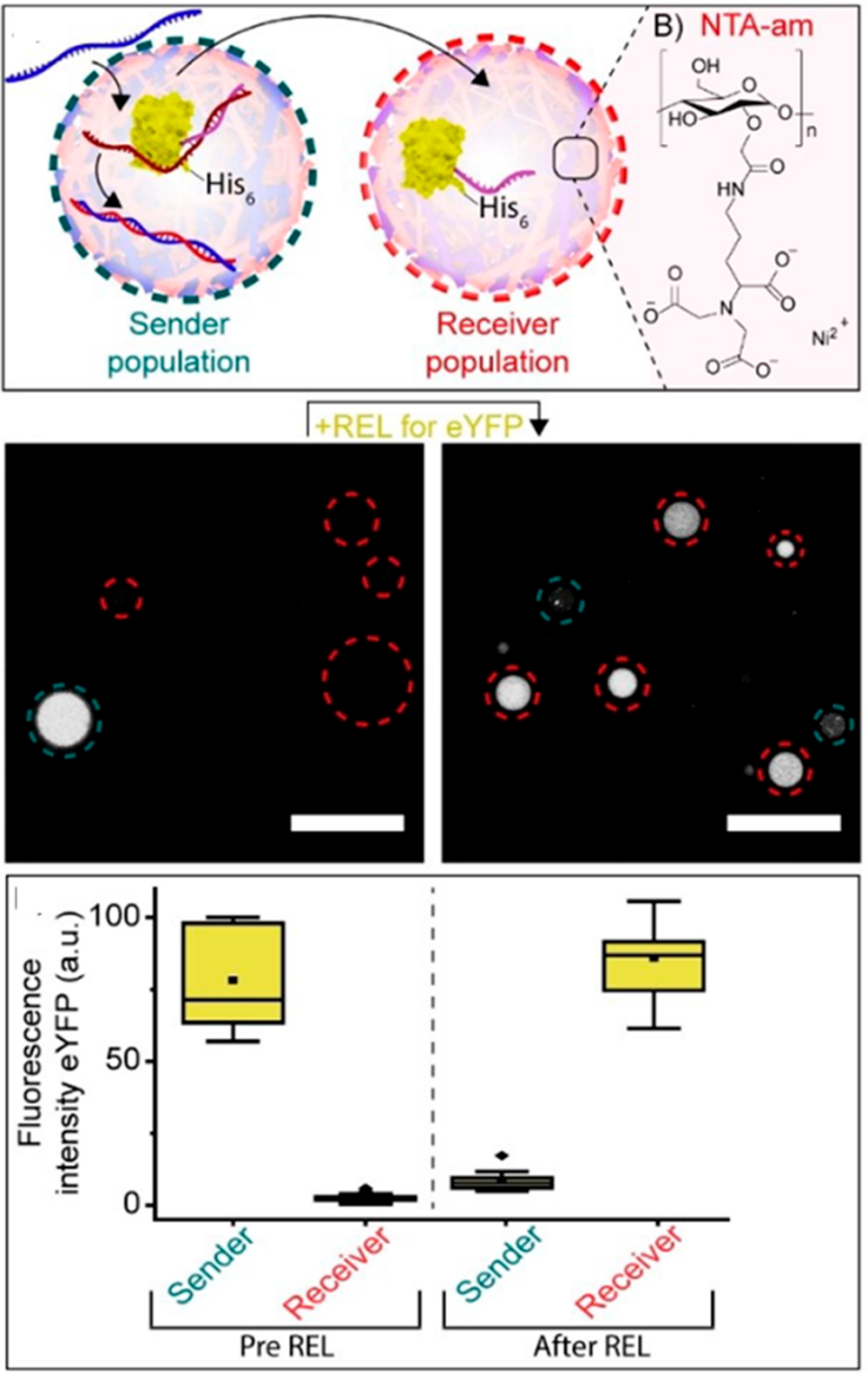
Protein-based shuttling between different populations of complex coacervate artificial cells. First population containing eYFP and a specific DNA strand, upon addition (and coacervate uptake) of a complementary DNA strand, the protein is excreted. eYFP is then taken up by the second population of artificial cells, as observed by confocal microscopy over 60 min. Reproduced with permission from ref (47). Copyright 2022 The Author(s).

So far many types of molecules have been investigated for artificial cell communications, including proteins, DNA, and small molecules (generated from enzyme-based cascade reactions). Our group has recently developed a protein shuttling system, where particular DNA strands are used as triggers to induce uptake and release of fluorescent proteins in two coacervate cell populations (Figure 6).^47^ This simplified signaling concept provides a method to engineer life-like communication pathways involving protein secretion, similarly to pathways in higher order multicellular constructs. Other systems have been developed focusing on other important biomolecular signals, such as DNA itself, to achieve information transfer in artificial cell populations. The group of de Greef in particular have utilized aqueous vesicles to achieve DNA strand communication via diffusion,^48^ while the group of Walther have employed DNA-based coacervates for similar DNA strand communication studies.^49^

**Figure 7:**
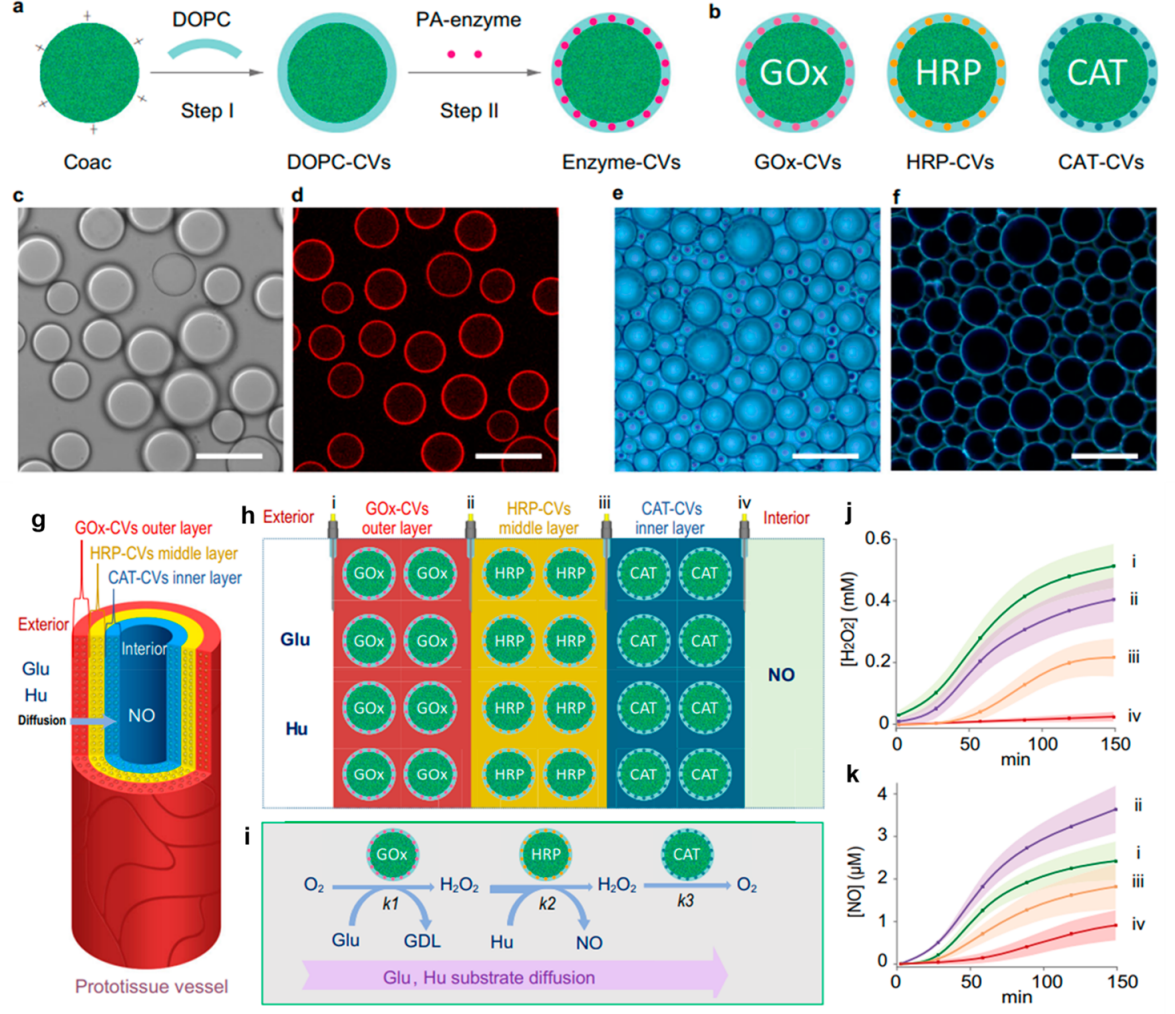
Enzyme decorated coacervate artificial cells able to process multiple signaling molecules. a) Formation of lipid membrane stabilized PDDA/DNA coacervate droplets and enzyme immobilization to achieve. b) Gox, HRP, and CAT coacervate droplets. c) Bright-field. d) Fluorescence. e) Optical phase contrast. f) Dark field microscopy images of complex coacervates showing continuous phospholipid membrane. g) Fabrication of tubular prototissue containing three artificial cell populations embedded in hydrogel. h), (i) Enzyme cascade reaction schematic showing positions of H_2_O_2_ and NO monitoring (i–iv) and substrate reaction diffusion process, j), k) H_2_O_2_ and NO were monitored over time with colorimetric reactions (ABTS oxidation, and Greiss reagent). Reproduced with permission from ref (50). Copyright 2022 The Author(s) distributed under a Creative Commons Attribution License 4.0 (CC BY-NC).

In the realm of small molecule signaling and communication, early work was carried out in lipid vesicles, where bacteria would produce a bioluminescent signal on sensing of a lipid vesicle synthesized sugar molecule.^51^ In polymer coacervate artificial cells, enzymes have been used to sense small molecules through production of fluorescent moieties able to be easily detected. This has been expanded recently by Liu et al., who showed that enzyme containing artificial cells could be built into tissue-like structures maintaining segregation of the coacervate populations (Figure 7).^50^ A tubular layered prototissue was used to generate NO inside the tube upon an external glucose/hydroxyurea signal (over 150 min the NO concentration internally was able to reach around 1 μM). The anticoagulation activity of the produced NO was then investigated by measuring real time light scattering of blood plasma, which in the NO producing device was dramatically reduced (*t*_1/2_ = 280 min) compared to the control non-NO producing device (*t*_1/2_ = 32 min).

This highlighted research in biomolecule and small molecule signaling in artificial cell systems represents an exciting direction for biomimetic materials and prototissue synthesis from a bottom-up strategy. However, there are still many unexplored research directions. One interesting example for artificial cell communication studies is the incorporation of further functional genetic material to create systems capable of collective behavior based on signaling with more life-like pathways.

### 3.4 Motility

Cell migration is fundamental to the development and health of living organisms. Aspects of tissue characteristics, such as wound healing, immune response, and developmental biology, all require cell movement. This cell motility can be determined by chemical or mechanical cues. Failure in aspects of these motile systems can lead to diseases including tumor growth, neurodevelopmental disorders, and vasculature diseases, therefore increasing understanding of cell motility could lead to new therapeutic opportunities.

Engineering the movement of artificial cells can be achieved not only with chemical fuels but also with magnetism, electric fields, and light. The latter three are interesting options because they can be triggered externally to potentially achieve some form of spatiotemporal control. Agrawal et al. used defined electric fields to investigate the movement of coacervate droplets both individually through a microfabricated path and collectively as groups of droplets in an oscillating AC field.^52^ The PDDA/ATP coacervates were stable to coalescence due to an interfacial increase in noncovalent interactions when the coacervates were transferred from buffer to deionized water. Movement of the charged droplets through a maze structure was performed with two orthogonal DC fields. Achieving motion through electric field of a noncovalent electrostatically assembled structure is noteworthy, as this has only been shown with rigid or cross-linked particles before. Interestingly, when an AC field was applied to a solution of coacervate droplets they formed aligned chain-like structures of connected yet distinct droplets, due to the polarizable nature of the phase separated droplets. This transient polarization of cell-like coacervate droplets will be useful as a model system for further understanding electrodynamics and hierarchical assembly of living systems.

**Figure 8:**
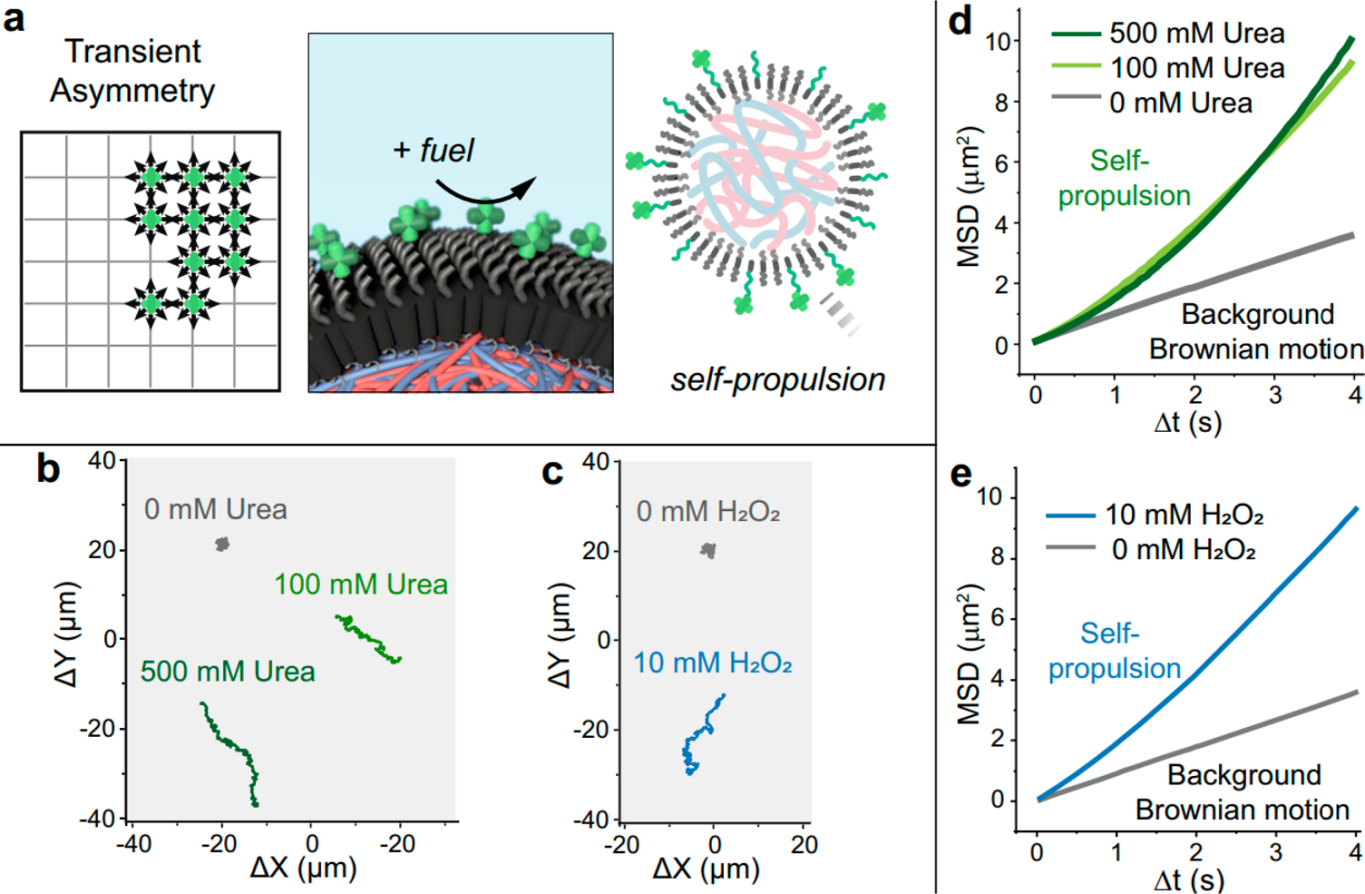
Enzyme motor driven motility of polymer coacervate droplets. a) Schematic diagram of transient asymmetry in enzyme distribution due to lateral diffusion in the membrane. b) Trajectories of urease or c) catalase motile artificial cells, fueled by the addition of urea or H_2_O_2_, respectively. d) Mean square displacement curves of urease and e) catalase motile artificial cells. Reproduced with permission from ref (53). Copyright 2021 The Author(s) distributed under a Creative Commons Attribution-Noncommercial License 4.0 (CC BY-NC).

Our group has also looked at the motility of coacervate artificial cells, using chemical fuels and enzymes as motors (Figure 8).^53^ Using bioorthogonal click chemistry, the enzymes urease and catalase were conjugated to the polymer membrane of the amylose coacervate system. Due to the fluid-like nature of the self-assembled polymer membrane, the enzymes were able to laterally diffuse, thus introducing transient asymmetry to the artificial cell surface configuration of enzymes. Chemical fuel mediated movement of the coacervate cell-like droplets was determined by a balance of enzyme density and transient asymmetry with medium densities having a higher probability of displaying asymmetric arrangement of surface enzymes. These recent examples highlight the use of electric fields and chemical fuels to drive the motion of coacervate artificial cells; other examples of noncoacervate systems having induced motion include systems propelled with light irradiation or are due to other physical phenomena such as Marangoni flows.^54^

### 3.5 Metabolism, Growth, and Division

Some of the most important aspects of cellular machinery are also the most complex and difficult to replicate in synthetic systems. Metabolism, the process of energy consumption and waste production, is vital to cells and relies on interconnected mechanisms of (macro)molecule production and degradation, in the presence of energy-rich phosphate compounds ATP/ADP. The reproduction of these complex pathways in artificial cells is challenging.^55^ Such a system would require a dynamic equilibrium to maintain the appropriate levels of nutrients as well as waste removal, in order to have continued metabolic processes over longer time scales. Linked to metabolism are the functions of growth and eventually division (in the case of a self-sustaining system).^56^ All of these dynamic functions have only been shown for polymer-based coacervate artificial cells in a few cases (although in cell-like GUV droplets there are more examples).^57,58^

**Figure 9:**
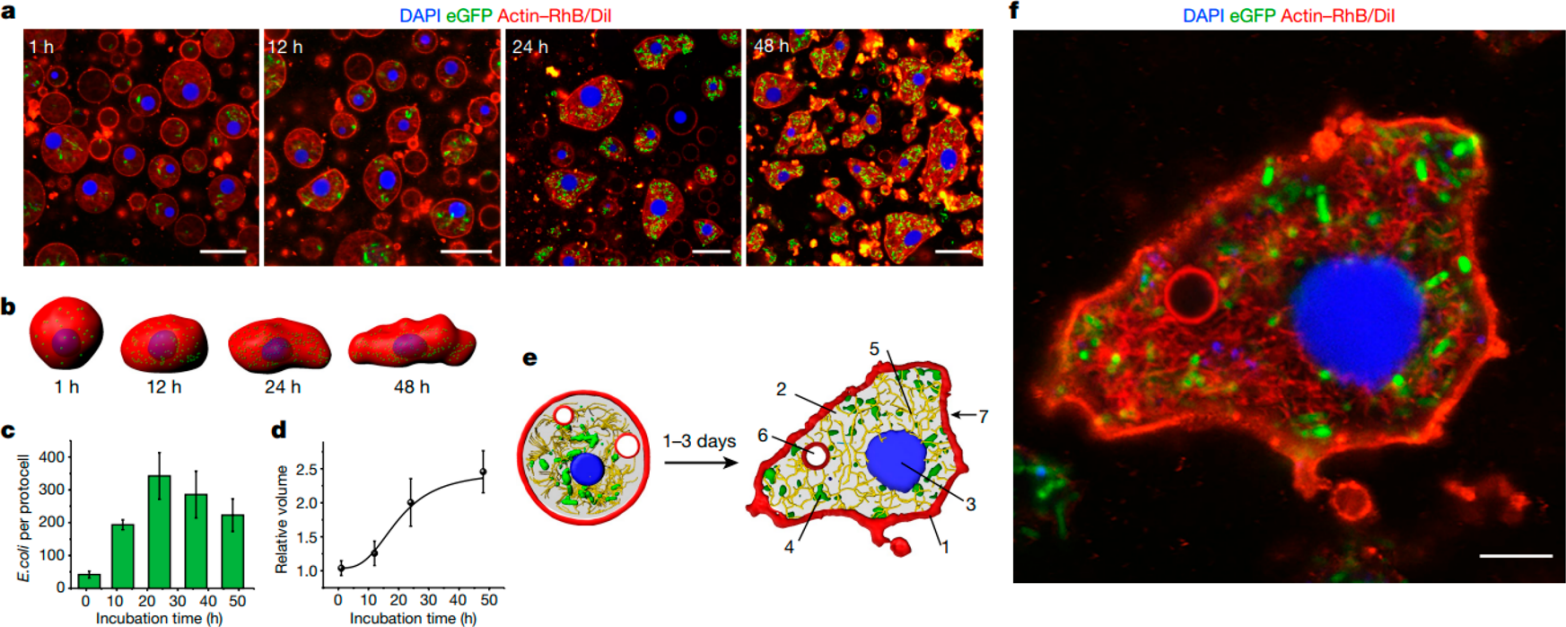
Assembly of live-cell containing polymer coacervate system and its dynamic morphogenesis. a) Confocal microscopy images over time showing transformation from spherical artificial cells to nonspherical. b) 3D reconstruction. c) Changes in number of live *E. coli* cells per artificial cell. d) Mean relative volumes of artificial cells over time. e) Diagram. f) Confocal micrograph of nonspherical artificial cell showing life-like features: (1) outer membrane, (2) crowded macromolecular cytoplasm, (3) DNA/Histone organelle, (4) encapsulated *E. coli* (mitochondria mimicking), (5) actin network, (6) spherical vacuoles, (7) amoeba-like morphology. Scale bars: 10 μm. Reproduced with permission from ref (14). Copyright 2022 The Authors.

Nakashima et al. investigated active coacervate protocells that were able to grow in size through a pyruvate kinase (PyK) catalyzed mechanism.^59^ Lysine rich protein K_72_ in the presence of ADP did not form droplets, but in the presence of PyK, added phosphoenol pyruvate was hydrolyzed to pyruvate and ATP was formed. The produced ATP was able to form coacervate droplets with K_72_, and the growth of droplets was controlled by the calculated addition of the fuel molecule phosphoenol pyruvate. The most advanced example of a dynamic and metabolic coacervate artificial cell to date was recently published by the Mann and Li groups (see Figure 9).^14^ The authors developed a multistep assembly process, featuring an outer lipid membrane from lysed cells, a molecularly crowded coacervate core, with a complex multiphase structure containing additional bacterial components and nucleic acids. The PDDA/ATP coacervate bacteriogenic protocells contained a DNA/histone/carboxymethyldextran phase separated nucleus-like structure inside the phase separated cell-like droplets. The system was able to produce ATP from live *E. coli* encapsulated in the droplets, which in turn could polymerize actin into a microfilament network. Finally, the authors also showed that, over time (48 h period), the protocells could not only grow in volume (2×) but also undergo morphogenesis, transforming from spherical to amoeba-like nonspherical shapes due to the internal metabolic activity.

## 4 Future Directions

The recent explosion of interest in liquid–liquid phase separation and biomolecular condensates in cells is increasing our understanding of the biophysics involved and our recognition of the importance of phase separation behavior in the origin of early cell structures. In the future, multidisciplinary teams of chemists, physicist, biologists, and materials scientists will be able to tackle more complex research questions involving coacervate-based artificial cells. The most exciting recent research in the artificial cell field, has involved integration of life-like functions such as dynamic metabolism and growth in micrometer-sized hierarchical materials. This will continue in the future with further integration of genetic information and IVTT machinery into coacervate droplets, which has to date been more challenging than their equivalent incorporation into aqueous droplets or GUVs. Achieving this could allow production of cytoskeletons or cell division machinery in situ, to further extend the lifetime of artificial cells. IVTT in coacervate artificial cells would also allow more complex communication based on nucleic acids and signaling proteins or cytokines. Additionally, the materials used for complex coacervates can, depending on the charged state, be toxic to cells. Future research will further develop these systems to be more biocompatible, while still maintaining enough charge density to form stable coacervate droplets.

Another particularly exciting avenue of research will be assembly of artificial cells into macroscopic materials with higher order structure. Some groups have already made early progress in this regard, for example, several groups have utilized 3D printing and other droplet manipulation techniques to fabricate artificial cells into 3D synthetic tissues or prototissues.^50,60^ Use of membrane stabilized coacervate artificial cells for the fabrication of tissues could open the door to a number of new applications in tissue regeneration and organoid formation. The controlled placement of artificial cells in 3D space could also allow for higher degrees of spatiotemporal control in the mimicking of functions such as communication. We think one of the most exciting advances in the field will come from the interaction of artificial cells with living cells to create hybrid materials with interactive properties. The assembly of interactive materials that have the ability to self-organize, can dynamically repair, and can also sense and respond to their biological surroundings will be a research target for the future. The interplay between artificial and living cells could lead to new applications in biomedicine, including new ways to modulate or restore diseased cell behavior and add or repair cell function. The isolation of particular cell-like characteristics *in vitro* also allows the study of various pharmaceutically relevant interactions in a life-like environment and could lead to artificial cell screening of drug compounds in the future. From a more industrial viewpoint, it is possible artificial cells could aid in the growing applications of synthetic biology for the advancement of sustainable manufacturing of chemicals, for agricultural applications, or in the food and drink industry.

## 5 Conclusions

In this Account, we have summarized new developments of synthetic and semisynthetic polymer materials in the mimicking of cellular systems with coacervate droplets. Fundamental aspects of coacervate formation were first discussed with a focus on the ionic interactions of complex coacervates systems. Factors affecting liquid–liquid phase separation were also outlined, and phase diagrams briefly highlighted as a convenient visual tool to understand whether a particular material and conditions will phase separate. The materials so far investigated as complex coacervate-forming artificial cell systems include charged synthetic polymers, modified polysaccharides, and polypeptides.

Second, with reference to leading examples from the literature and our group’s own research, we examined the key features of natural cells and how coacervate materials have been successful in mimicking these. Life-like artificial cell systems should ideally have viscoelastic mechanical properties, outer membranes, hierarchical compartmentalization, communicative features, motility, and possibly the ability to have self-sustaining functions like metabolism, growth, and division. Finally, we have mentioned some areas of research where coacervate-based artificial cells can have impact and advance the field of biomimetic systems, to achieve increasingly life-like materials.

## References

[ref1] Buddingh’ B. C.; van Hest J. C. M. Artificial Cells: Synthetic Compartments with Life-like Functionality and Adaptivity. Acc. Chem. Res. 2017, 50 (4), 769–777. 10.1021/acs.accounts.6b00512. 28094501 PMC5397886

[ref2] Qian X.; Nymann Westensee I.; Brodszkij E.; Städler B. Cell Mimicry as a Bottom-up Strategy for Hierarchical Engineering of Nature-Inspired Entities. WIREs Nanomedicine Nanobiotechnology 2021, 13 (3), e1683 10.1002/wnan.1683. 33205632

[ref3] van Stevendaal M. H. M. E.; van Hest J. C. M.; Mason A. F. Functional Interactions Between Bottom-Up Synthetic Cells and Living Matter for Biomedical Applications. ChemSystemsChem. 2021, 3 (5), e2100009 10.1002/syst.202100009.

[ref4] Hyman A. A.; Weber C. A.; Jülicher F. Liquid-Liquid Phase Separation in Biology. Annu. Rev. Cell Dev. Biol. 2014, 30 (1), 39–58. 10.1146/annurev-cellbio-100913-013325. 25288112

[ref5] Insua I.; Montenegro J. Synthetic Supramolecular Systems in Life-like Materials and Protocell Models. Chem. 2020, 6 (7), 1652–1682. 10.1016/j.chempr.2020.06.005.

[ref6] Tanwar L.; Devaraj N. K. Engineering Materials for Artificial Cells. Curr. Opin. Solid State Mater. Sci. 2022, 26 (4), 101004 10.1016/j.cossms.2022.101004.

[ref7] Göpfrich K.; Haller B.; Staufer O.; Dreher Y.; Mersdorf U.; Platzman I.; Spatz J. P. One-Pot Assembly of Complex Giant Unilamellar Vesicle-Based Synthetic Cells. ACS Synth. Biol. 2019, 8 (5), 937–947. 10.1021/acssynbio.9b00034. 31042361 PMC6528161

[ref8] Weiss M.; Frohnmayer J. P.; Benk L. T.; Haller B.; Janiesch J.-W.; Heitkamp T.; Börsch M.; Lira R. B.; Dimova R.; Lipowsky R.; Bodenschatz E.; Baret J.-C.; Vidakovic-Koch T.; Sundmacher K.; Platzman I.; Spatz J. P. Sequential Bottom-up Assembly of Mechanically Stabilized Synthetic Cells by Microfluidics. Nat. Mater. 2018, 17 (1), 89–96. 10.1038/nmat5005. 29035355

[ref9] Gonzales D. T.; Yandrapalli N.; Robinson T.; Zechner C.; Tang T.-Y. D. Cell-Free Gene Expression Dynamics in Synthetic Cell Populations. ACS Synth. Biol. 2022, 11 (1), 205–215. 10.1021/acssynbio.1c00376. 35057626 PMC8787815

[ref10] Allen M. E.; Hindley J. W.; Baxani D. K.; Ces O.; Elani Y. Hydrogels as Functional Components in Artificial Cell Systems. Nat. Rev. Chem. 2022, 6 (8), 562–578. 10.1038/s41570-022-00404-7. 37118012

[ref11] Zhou X.; Wu H.; Cui M.; Lai S. N.; Zheng B. Long-Lived Protein Expression in Hydrogel Particles: Towards Artificial Cells. Chem. Sci. 2018, 9 (18), 4275–4279. 10.1039/C8SC00383A. 29780558 PMC5944208

[ref12] Brangwynne C. P.; Tompa P.; Pappu R. V. Polymer Physics of Intracellular Phase Transitions. Nat. Phys. 2015, 11 (11), 899–904. 10.1038/nphys3532.

[ref13] Alberti S.; Gladfelter A.; Mittag T. Considerations and Challenges in Studying Liquid-Liquid Phase Separation and Biomolecular Condensates. Cell 2019, 176 (3), 419–434. 10.1016/j.cell.2018.12.035. 30682370 PMC6445271

[ref14] Xu C.; Martin N.; Li M.; Mann S. Living Material Assembly of Bacteriogenic Protocells. Nature 2022, 609 (7929), 1029–1037. 10.1038/s41586-022-05223-w. 36104562

[ref15] Choi S.; Meyer M. O.; Bevilacqua P. C.; Keating C. D. Phase-Specific RNA Accumulation and Duplex Thermodynamics in Multiphase Coacervate Models for Membraneless Organelles. Nat. Chem. 2022, 14 (10), 1110–1117. 10.1038/s41557-022-00980-7. 35773489

[ref16] Guindani C.; da Silva L. C.; Cao S.; Ivanov T.; Landfester K. Synthetic Cells: From Simple Bio-Inspired Modules to Sophisticated Integrated Systems. Angew. Chem., Int. Ed. 2022, 61 (16), e202110855 10.1002/anie.202110855. PMC9314110 34856047

[ref17] Sing C. E.; Perry S. L. Recent Progress in the Science of Complex Coacervation. Soft Matter 2020, 16 (12), 2885–2914. 10.1039/D0SM00001A. 32134099

[ref18] Oparin A. I. The Origin of Life; Dover, 1953.

[ref19] Mason A. F.; Buddingh’ B. C.; Williams D. S.; van Hest J. C. M. Hierarchical Self-Assembly of a Copolymer-Stabilized Coacervate Protocell. J. Am. Chem. Soc. 2017, 139 (48), 17309–17312. 10.1021/jacs.7b10846. 29134798 PMC5724030

[ref20] Dora Tang T.-Y.; Rohaida Che Hak C.; Thompson A. J.; Kuimova M. K.; Williams D. S.; Perriman A. W.; Mann S. Fatty Acid Membrane Assembly on Coacervate Microdroplets as a Step towards a Hybrid Protocell Model. Nat. Chem. 2014, 6 (6), 527–533. 10.1038/nchem.1921. 24848239

[ref21] Abbas M.; Lipiński W. P.; Wang J.; Spruijt E. Peptide-Based Coacervates as Biomimetic Protocells. Chem. Soc. Rev. 2021, 50 (6), 3690–3705. 10.1039/D0CS00307G. 33616129

[ref22] Black K. A.; Priftis D.; Perry S. L.; Yip J.; Byun W. Y.; Tirrell M. Protein Encapsulation via Polypeptide Complex Coacervation. ACS Macro Lett. 2014, 3 (10), 1088–1091. 10.1021/mz500529v. 35610798

[ref23] Abbas M.; Law J. O.; Grellscheid S. N.; Huck W. T. S.; Spruijt E. Peptide-Based Coacervate-Core Vesicles with Semipermeable Membranes. Adv. Mater. 2022, 34 (34), 2202913 10.1002/adma.202202913. 35796384

[ref24] Love C.; Steinkühler J.; Gonzales D. T.; Yandrapalli N.; Robinson T.; Dimova R.; Tang T.-Y. D. Reversible PH-Responsive Coacervate Formation in Lipid Vesicles Activates Dormant Enzymatic Reactions. Angew. Chem., Int. Ed. 2020, 59 (15), 5950–5957. 10.1002/anie.201914893. PMC7187140 31943629

[ref25] Cakmak F. P.; Choi S.; Meyer M. O.; Bevilacqua P. C.; Keating C. D. Prebiotically-Relevant Low Polyion Multivalency Can Improve Functionality of Membraneless Compartments. Nat. Commun. 2020, 11 (1), 5949 10.1038/s41467-020-19775-w. 33230101 PMC7683531

[ref26] Capasso Palmiero U.; Paganini C.; Kopp M. R. G.; Linsenmeier M.; Küffner A. M.; Arosio P. Programmable Zwitterionic Droplets as Biomolecular Sorters and Model of Membraneless Organelles. Adv. Mater. 2022, 34 (4), 2104837 10.1002/adma.202104837. 34664748

[ref27] Gucht J. v. d.; Spruijt E.; Lemmers M.; Cohen Stuart M. A. Polyelectrolyte Complexes: Bulk Phases and Colloidal Systems. J. Colloid Interface Sci. 2011, 361 (2), 407–422. 10.1016/j.jcis.2011.05.080. 21705008

[ref28] Cook A. B.; Decuzzi P. Harnessing Endogenous Stimuli for Responsive Materials in Theranostics. ACS Nano 2021, 15 (2), 2068–2098. 10.1021/acsnano.0c09115. 33555171 PMC7905878

[ref29] Rumyantsev A. M.; Jackson N. E.; Yu B.; Ting J. M.; Chen W.; Tirrell M. V.; de Pablo J. J. Controlling Complex Coacervation via Random Polyelectrolyte Sequences. ACS Macro Lett. 2019, 8 (10), 1296–1302. 10.1021/acsmacrolett.9b00494. 35651159

[ref30] Cook A. B.; Perrier S. Branched and Dendritic Polymer Architectures: Functional Nanomaterials for Therapeutic Delivery. Adv. Funct. Mater. 2020, 30 (2), 1901001 10.1002/adfm.201901001.

[ref31] Cook A. B.; Peltier R.; Barlow T. R.; Tanaka J.; Burns J. A.; Perrier S. Branched Poly (Trimethylphosphonium Ethylacrylate-Co-PEGA) by RAFT: Alternative to Cationic Polyammoniums for Nucleic Acid Complexation. J. Interdiscip. Nanomedicine 2018, 3 (4), 164–174. 10.1002/jin2.50. PMC6360508 30774985

[ref32] Chang L.-W.; Lytle T. K.; Radhakrishna M.; Madinya J. J.; Vélez J.; Sing C. E.; Perry S. L. Sequence and Entropy-Based Control of Complex Coacervates. Nat. Commun. 2017, 8 (1), 1273 10.1038/s41467-017-01249-1. 29097695 PMC5668414

[ref33] Iglesias-Artola J. M.; Drobot B.; Kar M.; Fritsch A. W.; Mutschler H.; Dora Tang T.-Y.; Kreysing M. Charge-Density Reduction Promotes Ribozyme Activity in RNA–Peptide Coacervates via RNA Fluidization and Magnesium Partitioning. Nat. Chem. 2022, 14 (4), 407–416. 10.1038/s41557-022-00890-8. 35165426 PMC8979813

[ref34] Cook A. B.; Peltier R.; Zhang J.; Gurnani P.; Tanaka J.; Burns J. A.; Dallmann R.; Hartlieb M.; Perrier S. Hyperbranched Poly(Ethylenimine-Co-Oxazoline) by Thiol–Yne Chemistry for Non-Viral Gene Delivery: Investigating the Role of Polymer Architecture. Polym. Chem. 2019, 10 (10), 1202–1212. 10.1039/C8PY01648H.

[ref35] Podolsky K. A.; Devaraj N. K. Synthesis of Lipid Membranes for Artificial Cells. Nat. Rev. Chem. 2021, 5 (10), 676–694. 10.1038/s41570-021-00303-3. 37118179

[ref36] Huang X.; Li M.; Green D. C.; Williams D. S.; Patil A. J.; Mann S. Interfacial Assembly of Protein–Polymer Nano-Conjugates into Stimulus-Responsive Biomimetic Protocells. Nat. Commun. 2013, 4 (1), 2239 10.1038/ncomms3239. 23896993

[ref37] Cook A. B.; Schlich M.; Manghnani P. N.; Moore T. L.; Decuzzi P.; Palange A. L. Size Effects of Discoidal PLGA Nanoconstructs in Pickering Emulsion Stabilization. J. Polym. Sci. 2022, 60 (9), 1480–1491. 10.1002/pol.20210748.

[ref38] Koga S.; Williams D. S.; Perriman A. W.; Mann S. Peptide-Nucleotide Microdroplets as a Step towards a Membrane-Free Protocell Model. Nat. Chem. 2011, 3 (9), 720–724. 10.1038/nchem.1110. 21860462

[ref39] Liu S.; Zhang Y.; Li M.; Xiong L.; Zhang Z.; Yang X.; He X.; Wang K.; Liu J.; Mann S. Enzyme-Mediated Nitric Oxide Production in Vasoactive Erythrocyte Membrane-Enclosed Coacervate Protocells. Nat. Chem. 2020, 12 (12), 1165–1173. 10.1038/s41557-020-00585-y. 33219364

[ref40] Pir Cakmak F.; Marianelli A. M.; Keating C. D. Phospholipid Membrane Formation Templated by Coacervate Droplets. Langmuir 2021, 37 (34), 10366–10375. 10.1021/acs.langmuir.1c01562. 34398617

[ref41] Booth R.; Qiao Y.; Li M.; Mann S. Spatial Positioning and Chemical Coupling in Coacervate-in-Proteinosome Protocells. Angew. Chem., Int. Ed. 2019, 58 (27), 9120–9124. 10.1002/anie.201903756. PMC6618027 31034692

[ref42] Mason A. F.; Yewdall N. A.; Welzen P. L. W.; Shao J.; van Stevendaal M.; van Hest J. C. M.; Williams D. S.; Abdelmohsen L. K. E. A. Mimicking Cellular Compartmentalization in a Hierarchical Protocell through Spontaneous Spatial Organization. ACS Cent. Sci. 2019, 5 (8), 1360–1365. 10.1021/acscentsci.9b00345. 31482118 PMC6716124

[ref43] Karoui H.; Seck M. J.; Martin N. Self-Programmed Enzyme Phase Separation and Multiphase Coacervate Droplet Organization. Chem. Sci. 2021, 12 (8), 2794–2802. 10.1039/D0SC06418A. 34164043 PMC8179374

[ref44] Chen Y.; Yuan M.; Zhang Y.; Liu S.; Yang X.; Wang K.; Liu J. Construction of Coacervate-in-Coacervate Multi-Compartment Protocells for Spatial Organization of Enzymatic Reactions. Chem. Sci. 2020, 11 (32), 8617–8625. 10.1039/D0SC03849K. 34123122 PMC8163383

[ref45] Lu T.; Spruijt E. Multiphase Complex Coacervate Droplets. J. Am. Chem. Soc. 2020, 142 (6), 2905–2914. 10.1021/jacs.9b11468. 31958956 PMC7020193

[ref46] Mukwaya V.; Mann S.; Dou H. Chemical Communication at the Synthetic Cell/Living Cell Interface. Commun. Chem. 2021, 4 (1), 161 10.1038/s42004-021-00597-w. 36697795 PMC9814394

[ref47] Mashima T.; van Stevendaal M. H. M. E.; Cornelissens F. R. A.; Mason A. F.; Rosier B. J. H. M.; Altenburg W. J.; Oohora K.; Hirayama S.; Hayashi T.; van Hest J. C. M.; Brunsveld L. DNA-Mediated Protein Shuttling between Coacervate-Based Artificial Cells. Angew. Chem., Int. Ed. 2022, 61 (17), e202115041 10.1002/anie.202115041. PMC9303767 35133040

[ref48] Joesaar A.; Yang S.; Bögels B.; van der Linden A.; Pieters P.; Kumar B. V. V. S. P.; Dalchau N.; Phillips A.; Mann S.; de Greef T. F. A. DNA-Based Communication in Populations of Synthetic Protocells. Nat. Nanotechnol. 2019, 14 (4), 369–378. 10.1038/s41565-019-0399-9. 30833694 PMC6451639

[ref49] Samanta A.; Hörner M.; Liu W.; Weber W.; Walther A. Signal-Processing and Adaptive Prototissue Formation in Metabolic DNA Protocells. Nat. Commun. 2022, 13 (1), 3968 10.1038/s41467-022-31632-6. 35803944 PMC9270428

[ref50] Liu S.; Zhang Y.; He X.; Li M.; Huang J.; Yang X.; Wang K.; Mann S.; Liu J. Signal Processing and Generation of Bioactive Nitric Oxide in a Model Prototissue. Nat. Commun. 2022, 13 (1), 5254 10.1038/s41467-022-32941-6. 36068269 PMC9448809

[ref51] Gardner P. M.; Winzer K.; Davis B. G. Sugar Synthesis in a Protocellular Model Leads to a Cell Signalling Response in Bacteria. Nat. Chem. 2009, 1 (5), 377–383. 10.1038/nchem.296. 21378891

[ref52] Agrawal A.; Douglas J. F.; Tirrell M.; Karim A. Manipulation of Coacervate Droplets with an Electric Field. Proc. Natl. Acad. Sci. U. S. A. 2022, 119 (32), e2203483119 10.1073/pnas.2203483119. 35925890 PMC9372540

[ref53] Song S.; Mason A. F.; Post R. A. J.; De Corato M.; Mestre R.; Yewdall N. A.; Cao S.; van der Hofstad R. W.; Sanchez S.; Abdelmohsen L. K. E. A.; van Hest J. C. M. Engineering Transient Dynamics of Artificial Cells by Stochastic Distribution of Enzymes. Nat. Commun. 2021, 12 (1), 6897 10.1038/s41467-021-27229-0. 34824231 PMC8617035

[ref54] Haller B.; Jahnke K.; Weiss M.; Göpfrich K.; Platzman I.; Spatz J. P. Autonomous Directional Motion of Actin-Containing Cell-Sized Droplets. Adv. Intell. Syst. 2021, 3 (5), 2000190 10.1002/aisy.202000190.

[ref55] Olivi L.; Berger M.; Creyghton R. N. P.; De Franceschi N.; Dekker C.; Mulder B. M.; Claassens N. J.; ten Wolde P. R.; van der Oost J. Towards a Synthetic Cell Cycle. Nat. Commun. 2021, 12 (1), 4531 10.1038/s41467-021-24772-8. 34312383 PMC8313558

[ref56] Zwicker D.; Seyboldt R.; Weber C. A.; Hyman A. A.; Jülicher F. Growth and Division of Active Droplets Provides a Model for Protocells. Nat. Phys. 2017, 13 (4), 408–413. 10.1038/nphys3984.

[ref57] Bergmann A. M.; Donau C.; Späth F.; Jahnke K.; Göpfrich K.; Boekhoven J. Evolution and Single-Droplet Analysis of Fuel-Driven Compartments by Droplet-Based Microfluidics. Angew. Chem., Int. Ed. 2022, 61 (32), e202203928 10.1002/anie.202203928. PMC9400878 35657164

[ref58] Lewis R. W.; Klemm B.; Macchione M.; Eelkema R. Fuel-Driven Macromolecular Coacervation in Complex Coacervate Core Micelles. Chem. Sci. 2022, 13 (16), 4533–4544. 10.1039/D2SC00805J. 35656128 PMC9019912

[ref59] Nakashima K. K.; van Haren M. H. I.; André A. A. M.; Robu I.; Spruijt E. Active Coacervate Droplets Are Protocells That Grow and Resist Ostwald Ripening. Nat. Commun. 2021, 12 (1), 3819 10.1038/s41467-021-24111-x. 34155210 PMC8217494

[ref60] Cook A. B.; Clemons T. D. Bottom-Up versus Top-Down Strategies for Morphology Control in Polymer-Based Biomedical Materials. Adv. NanoBiomed Res. 2022, 2, 2100087 10.1002/anbr.202100087.

